# Rapid differentiation of *Xihuangcao* from the three *Isodon* species by UPLC-ESI-QTOF-MS/MS and chemometrics analysis

**DOI:** 10.1186/s13020-016-0120-y

**Published:** 2016-12-15

**Authors:** Lai Lai Wong, Zhitao Liang, Hubiao Chen, Zhongzhen Zhao

**Affiliations:** 1 School of Chinese Medicine, Hong Kong Baptist University, Kowloon, Hong Kong Special Administrative Region People’s Republic of China; 2 Research Center for Pharmacognosy, Institute of Chinese Materia Medica, Academy of Chinese Medical Sciences, Beijing, People’s Republic of China

## Abstract

**Background:**

*Isodon lophanthoides*, *I. lophanthoides* var. *graciliflorus* and *I. serra* are the three botanical sources of *Xihuangcao*, which are often used indiscriminately in herbal products. The aim of this study was to develop a rapid and accurate analytical method to identify the three different botanical sources of *Xihuangcao* by combining UPLC-ESI-QTOF-MS with chemometrics analysis.

**Methods:**

Fifteen batches of plants were collected as reference materials and their chemical profiles were analyzed by UPLC-ESI-QTOF-MS. These data were subsequently processed by statistical methods, including principal component analysis (PCA), hierarchical cluster analysis (HCA) and orthogonal partial least squared discriminant analysis (OPLS-DA). An automated sample class prediction model was also built using Naive Bayes as a class prediction algorithm to rapidly determine the source species of twenty-seven batches of commercial *Xihuangcao* samples.

**Results:**

The base peak chromatograms of the three authenticated species showed different patterns and twenty-seven peaks were chosen, including six diterpenoids, one phenolic acid and two glycosides to distinguish among these three species. The results showed good differentiation among the three species by PCA, HCA and OPLS-DA. *Isodon lophanthoides* var. *graciliflorus* was found to be the major botanical source of the commercial samples.

**Conclusion:**

UPLC-ESI-QTOF-MS and subsequent chemometrics analysis were demonstrated effective to differentiate among the three different species of plants used as *Xihuangcao*.

**Electronic supplementary material:**

The online version of this article (doi:10.1186/s13020-016-0120-y) contains supplementary material, which is available to authorized users.

## Background

*Xihuangcao* is a folk medicine that is commonly used in southern China as a *dampness*-draining, antiicteric and liver protection herb [1]. Furthermore, clinical research has highlighted the beneficial effects of *Xihuangcao* for the treatment of hepatitis with jaundice, leading to the development and commercialization of numerous proprietary medicines and functional food products based on *Xihuangcao* [1]. Four different plants have been recorded as sources of *Xihuangcao*, including *Isodon lophanthoides* (Buch.-Ham. ex D. Don) Hara (IL), *I. lophanthoides* var. *graciliflorus* (Benth.) H. Hara (ILG), *I. lophanthoides* var. *gerardianus* (Benth.) H. Hara and *I. serra* (Maxim.) Kudo (IS) [2]. According to a taxonomic revision, the plants in this genus have been much confused, especially for the varieties of *I. lophanthoides*. A new classification of *I. lophanthoides* and its varieties was suggested in 2004, in which *I. lophanthoides* var. *gerardianus* and *I. lophanthoides* var. *graciliflorus* were merged as *I. lophanthoides* var. *graciliflorus* (ILG) [3]. However, some experts believe that IS should not be used as *Xihuangcao* because its leaves do not have yellow juice when they are rubbed, which is recorded as a major characteristic of *Xihuangcao* in several ancient herbal medicine books [4]. Recent studies have also demonstrated the chemical compositions of IL, ILG and IS are different, which diterpenoids in IL and ILG are mainly abietane and tricyclic types and that of IS are *ent*-kaurane type; thus, they should not be used as one herb [1, 4].

Although *Xihuangcao* has multiple botanical sources, most of the *Xihuangcao*-based products do not specify the species used. The development of an analytical method to identify the three different source species of *Xihuangcao* is therefore strongly desired to ensure the safe and effective use of these herbal products. IL and its variety ILG are difficult to distinguish based on their morphological or microscopic features [5, 6]. In contrast, IS can be readily distinguished from IL and ILG based on its microscopic features, but this method cannot be applied to differentiate between samples of different sources in their extracted forms. It is noteworthy that *Xihuangcao* has not yet been recorded in *The Pharmacopoeia of the People’s Republic of China*. The only official method available for the analysis of *Xihuangcao* is recorded in the *Guangdong Chinese Materia Medica Standards*. According to this method, samples for analysis are compared with a reference material (*I. lophanthoides* var. *graciliflorus*) by thin-layer chromatography with no provision for differentiating between the three species [2]. Although numerous studies have been conducted in recent years concerning *Xihuangcao*, most of these studies have limited to the chemical separation or pharmacological evaluation [7–10]. A few chemical analytical studies have been published, but each study analyzed a single species only [11, 12].

Chromatographic fingerprinting is an effective analytical method for identifying samples based on a comparison of their chemical profiles. Ultra-performance liquid chromatography (UPLC) can be used to separate a wide variety of compounds with superior resolution over a short period of time, and can also be coupled with mass spectrometry (MS). The major drawback of this method is that it can be time consuming to analyze all the MS data generated from a large number of samples. Chemometrics solves this problem by statistical analysis to distinguish between different species, thereby avoiding the manual comparison of huge amounts of raw MS data. Furthermore, class prediction models, which can be built based on the statistical interpretation of known reference samples can be used for automated classification of unknown samples.

The aim of this study was to identify the three botanical sources of *Xihuangcao* by ultra-performance liquid chromatography coupled with electrospray ionization quadrupole time-of-flight mass spectrometry (UPLC-ESI-QTOF-MS) in combination with chemometrics analysis.

## Methods

### Chemicals and reference standards

All chemicals and reference standards used in this study were purchased as the HPLC grade. Oridonin (purity ≥98%) and rosmarinic acid (purity ≥98%) were purchased from Chengdu Must Bio-Technology Co., Ltd (Chengdu, China). Schaftoside (purity ≥98%) was purchased from Chengdu Biopurify Phytochemicals Ltd (Chengdu, China). Methanol and acetonitrile were purchased from E. Merck (Darmstadt, Germany). Formic acid (purity 96.0%) was purchased from Tedia Company Inc. (Fairfield, OH, USA). Water was obtained from a Milli-Q water purification system (Millipore, Bedford, MA, USA).

### Plant materials and sample preparation

**Fig. 1:**
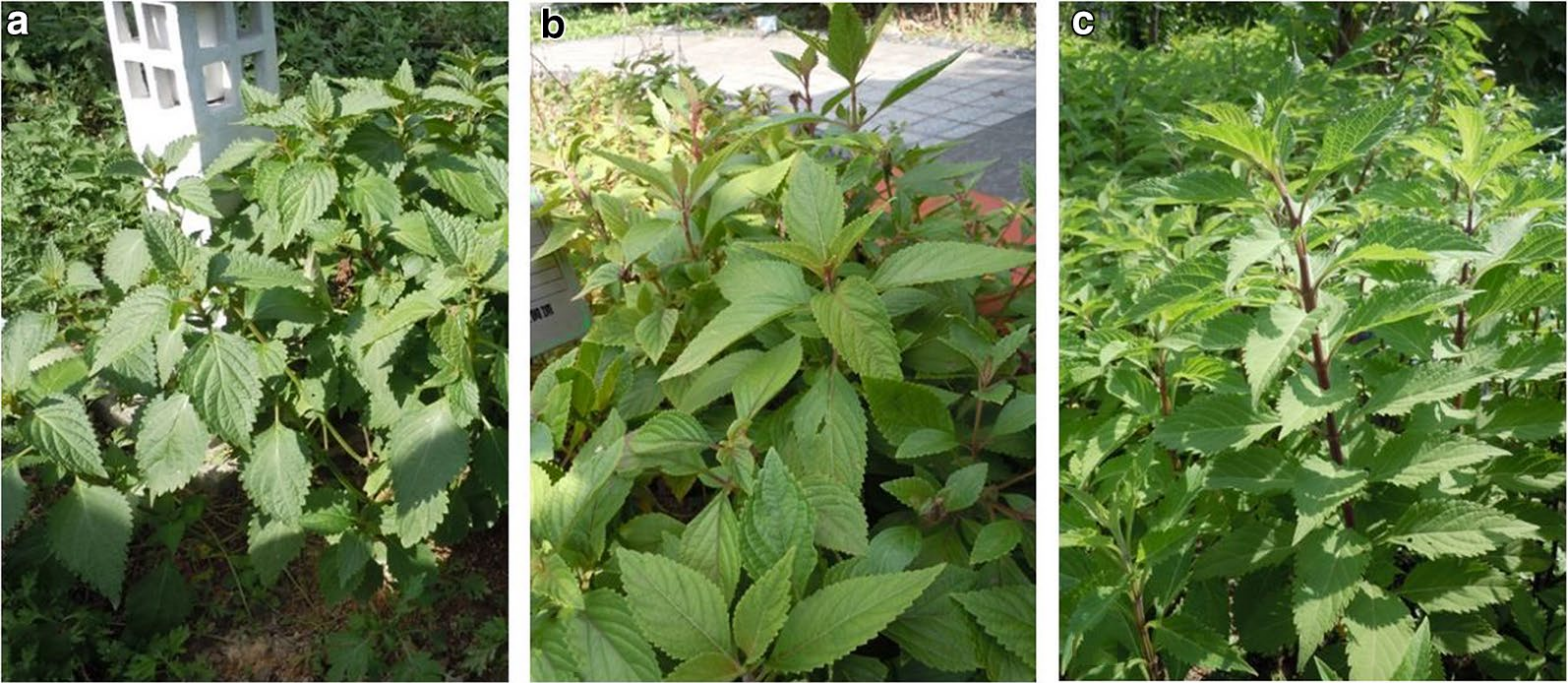
The botanical sources of *Xihuangcao*: **a** *I. lophanthoides*, **b** *I. lophanthoides* var. *graciliflorus* and **c** *I. serra*

**Fig. 2:**
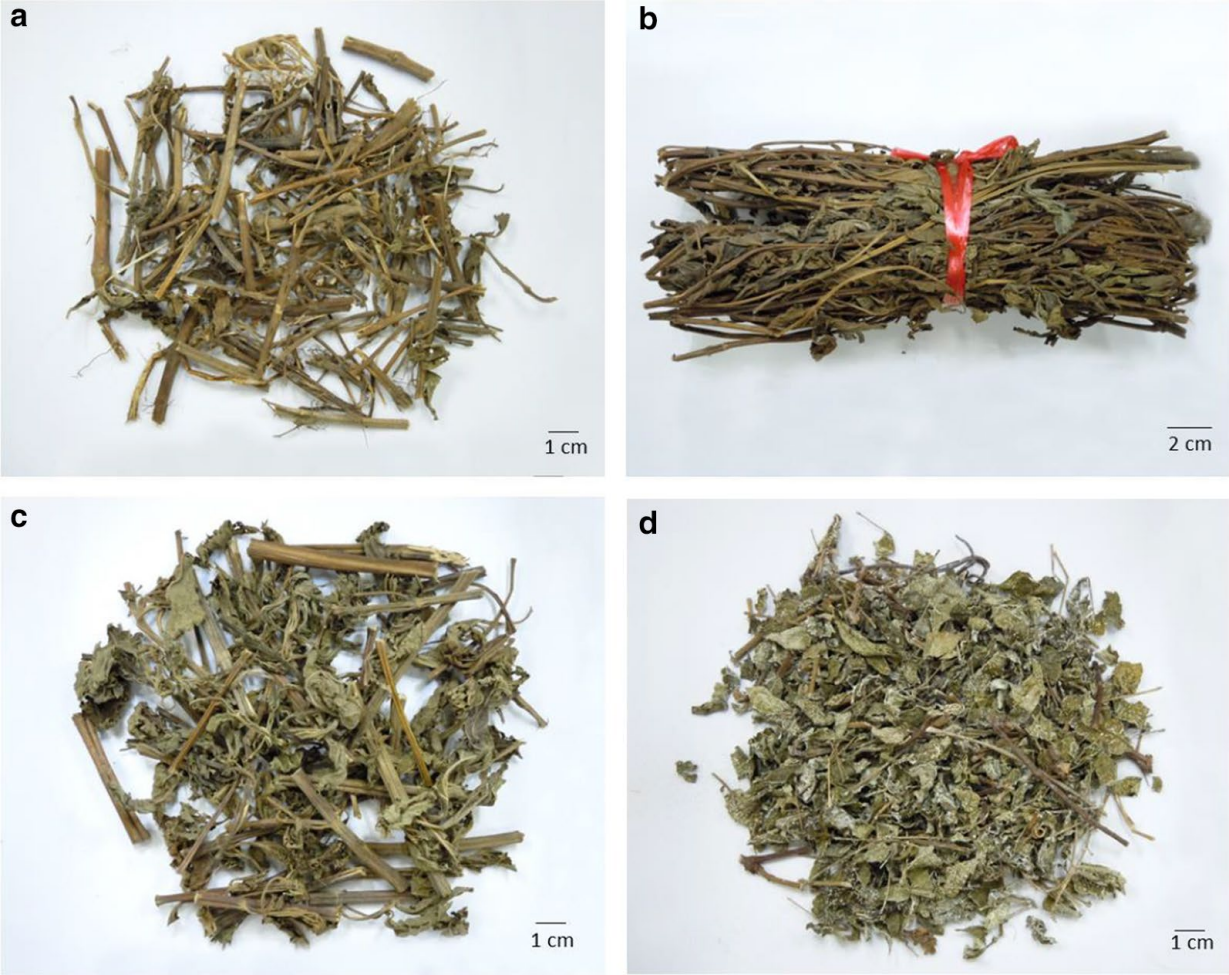
Photos of some of the commercial *Xihuangcao* samples collected in this study: **a**, **b** *I. lophanthoides* var. *graciliflorus,* **c** *I. serra* and **d** *Ampelopsis grossedentata*

Fifteen batches of plant samples were collected in southern China as reference materials, including four batches of *I. lophanthoides* var*. lophanthoides,* seven batches of *I. lophanthoides* var. *graciliflorus* and four batches of *I. serra* (Table 1; Fig. 1). These samples were authenticated by Prof. Zhongzhen Zhao (School of Chinese Medicine, Hong Kong Baptist University, Hong Kong) based on their morphological characteristics [3, 13]. Twenty-seven samples of commercial batches of *Xihuangcao* were collected from retail markets in Guangdong and Guangxi (Table 2; Fig. 2).

**Table 1:** Plant samples of *Xihuangcao* collected

Sample no.	Species	Source	Date of collection
IL-01	*Isodon lophanthoides*	Baiyun, Guangdong Province (transplant from Fujian)	July 2012
IL-02	*Isodon lophanthoides*	Baiyun, Guangdong Province	July 2012
IL-03	*Isodon lophanthoides*	Yingde, Guangdong Province	July 2012
IL-04	*Isodon lophanthoides*	Yingde, Guangdong Province	July 2012
ILG-01	*Isodon lophanthoides* var. *graciliflora*	Pingyuan, Guangdong Province	July 2012
ILG-02	*Isodon lophanthoides* var. *graciliflora*	Pingyuan, Guangdong Province	July 2012
ILG-03	*Isodon lophanthoides* var. *graciliflora*	Heping, Guangdong Province	July 2012
ILG-04	*Isodon lophanthoides* var. *graciliflora*	Baiyun, Guangdong Province	July 2012
ILG-04	*Isodon lophanthoides* var. *graciliflora*	Shatin, Hong Kong	August 2013
ILG-05	*Isodon lophanthoides* var. *graciliflora*	Meizhou, Guangdong Province	September 2013
ILG-06	*Isodon lophanthoides* var. *graciliflora*	Panyu, Guangdong Province	February 2014
IS-01	*Isodon serra*	Yingde, Guangdong Province	July 2012
IS-02	*Isodon serra*	Baiyun, Guangdong Province	July 2012
IS-03	*Isodon serra*	Zhaoqing, Guangdong Province	July 2012
IS-04	*Isodon serra*	Panyu, Guangdong Province	February 2014

**Table 2:** Commercial *Xihuangcao* samples collected and the identification result

Sample no.	**Producing area**	**Usage**	**Species identified**
Sample 01	Shangsha, Guangdong	Ingredient of soup	*Isodon lophanthoides* var. *graciliflorus*
Sample 02	Meizhou, Guangdong	Ingredient of soup	*Isodon lophanthoides* var. *graciliflorus*
Sample 03	Guangxi	Herb tea	*Ampelopsis grossedentata*
Sample 04	Huizhou, Guangdong	Herb tea	*Ampelopsis grossedentata*
Sample 05	Yulin, Guangxi	Decoction pieces	*Isodon serra*
Sample 06	Yulin, Guangxi	Herb tea	*Ampelopsis grossedentata*
Sample 07	Shaoguan, Guangdong	Herb tea	*Isodon serra*
Sample 08	Meizhou, Guangdong	Ingredient of soup	*Isodon lophanthoides* var. *graciliflorus*
Sample 09	Taiping, Guangxi	Herb tea	*Ampelopsis grossedentata*
Sample 10	Wengyuan, Guangdong	Herb tea	*Isodon serra*
Sample 11	Wengyuan, Guangdong	Herb tea	*Ampelopsis grossedentata*
Sample 12	Guangdong	Herb tea	*Isodon serra*
Sample 13	Meizhou, Guangdong	Ingredient of soup	*Isodon lophanthoides* var. *graciliflorus*
Sample 14	Heyuan, Guangdong	Herb tea	*Ampelopsis grossedentata*
Sample 15	Guangdong	Herb tea	*Ampelopsis grossedentata*
Sample 16	Meizhou, Guangdong	Ingredient of soup	*Isodon lophanthoides* var. *graciliflorus*
Sample 17	Meizhou, Guangdong	Ingredient of soup	Unknown
Sample 18	Jiangxi	Decoction pieces	*Isodon serra*
Sample 19	Meizhou, Guangdong	Ingredient of soup	*Isodon lophanthoides* var. *graciliflorus*
Sample 20	Huizhou, Guangdong	Herb tea	*Ampelopsis grossedentata*
Sample 21	Meizhou, Guangdong	Ingredient of soup	*Isodon lophanthoides* var. *graciliflorus*
Sample 22	Meizhou, Guangdong	Ingredient of soup	*Isodon lophanthoides* var. *graciliflorus*
Sample 23	Longyan, Fujian	Herb tea	*Isodon lophanthoides* var. *graciliflorus*
Sample 24	Meizhou, Guangdong	Ingredient of soup	*Isodon lophanthoides* var. *graciliflorus*
Sample 25	Meizhou, Guangdong	Ingredient of soup	*Isodon lophanthoides* var. *graciliflorus*
Sample 26	Meizhou, Guangdong	Decoction pieces	*Isodon serra*
Sample 27	Meizhou, Guangdong	Decoction pieces	*Isodon lophanthoides* var. *graciliflorus*

For the preparation of the reference materials, the roots were removed from the plants together with any foreign matter, and the remaining materials were dried under the sun. All of the dried samples were then powdered in a blender. A sample (0.5 g) of the powdered material was then accurately weighed into a centrifuge tube followed by 10 mL of methanol, and the resulting mixture was sonicated for 30 min at room temperature. The supernatant was then centrifuged at 25,920×*g* for 10 min on an Eppendorf 5810 centrifuge (Eppendorf, Hamburg, Germany). The extract (0.2 mL) was then diluted with 0.8 mL of methanol and transferred into a brown HPLC vial for injection.

### UPLC-ESI-QTOF-MS analysis

UPLC-ESI-QTOF-MS analysis was performed on an Agilent 6540 accurate mass Q-TOF LC/MS system (Agilent Technologies, Santa Clara, CA, USA). Chromatography was performed on a UPLC C_18_ analytical column (2.1 ×  5 mm, I.D. 1.7 μm, ACQUITY UPLC^®^ BEH, Waters, Milford, MA, USA). The mobile phase used for the elution of the column consisted of 0.1% (v/v) formic acid in water (solvent A) and 0.1% (v/v) formic acid in acetonitrile (solvent B). A gradient elution was performed as follows: 0–15 min, 10–45% B; 15–23 min, 45–70% B; 23–25 min, 70–100% B. The flow rate was set at 0.4 mL/min with an injection volume of 2 μL, whilst the column temperature was maintained at 40 °C.

Detection by ESI-QTOF-MS/MS was performed in positive ion mode. The source parameters were set as follows: gas temperature, 300 °C; gas flow, 8.0 L/min; nebulizer pressure, 45 psi; sheath gas temperature, 350 °C; sheath gas flow, 8.0 L/min. The scan source parameters were set as follows: VCap, 3500; nozzle voltage, 500 V; fragmentation voltage, 120 V. Reference masses were used at *m/z* 121.0508 (purine) and 922.0097 (hexakisphosphazine). Automatic MS/MS was performed using a fixed collision energy of 15 eV.

### Qualitative analysis

Data were analyzed using the Agilent MassHunter Workstation Software-Qualitative Analysis software (version B.06.00, build 6.0.633.0, Agilent Technologies Inc., 2012) with the following settings: extraction restrict retention time, 2–25 min; peak height, ≥100 counts; charge state, 1; peck spacing tolerance, 0.0025 *m/z* plus 7.0 ppm; compound relative height,   ≥2.5%; absolute height,   ≥2000 counts; elements of C, H, O from 3 to 60, 0 to 120 and 0 to 30, respectively, for generating the formulae.

### Statistical analysis

Statistical analyses and class prediction of the UPLC-MS/MS results were carried out using the Agilent MassHunter Mass Profiler Professional Software (version B.02.02, Agilent Technologies Inc., 2011). Data were initially exported from the Mass Hunter Workstation before being imported into the Mass Profiler Professional Software with the following settings: abundance filtering with minimum absolute abundance, 5000 counts; all charge states permitted; retention time (RT) correction with a maximum of 0.5% plus 0.5 min; mass window 5.0 ppm plus 2.0 mDa; compound alignment with RT window 0.1% plus 0.15 min. One-way analysis of variance (ANOVA) was used with a *P* value of 0.05 to further filter these data. A 3D principal component analysis (PCA) was conducted on all of the samples. A hierarchical cluster analysis (HCA) was also conducted with the following settings: cluster on conditions, Euclidean as the distance metric, centroid as the linkage rule. An automated sample class prediction model was built based on the UPLC-MS/MS results of the reference plants with the following settings: Naive Bayes as the class prediction algorithm; validation type, N-fold; number of folds, 3; number of repeats, 10.

SIMCA (version 13.0.3.0, Umetrics AB 2013) was used to analyze selected peaks. The peak volumes were manually imported as X-variables, with the sample number as a primary ID, peak number as a secondary ID and sample speciation as a class ID. The model type was set using orthogonal partial least squared discriminant analysis (OPLS-DA), where the significant components were calculated using an “Autofit” function.

## Results and discussion

### Chromatographic profiling of the reference plants

**Fig. 3:**
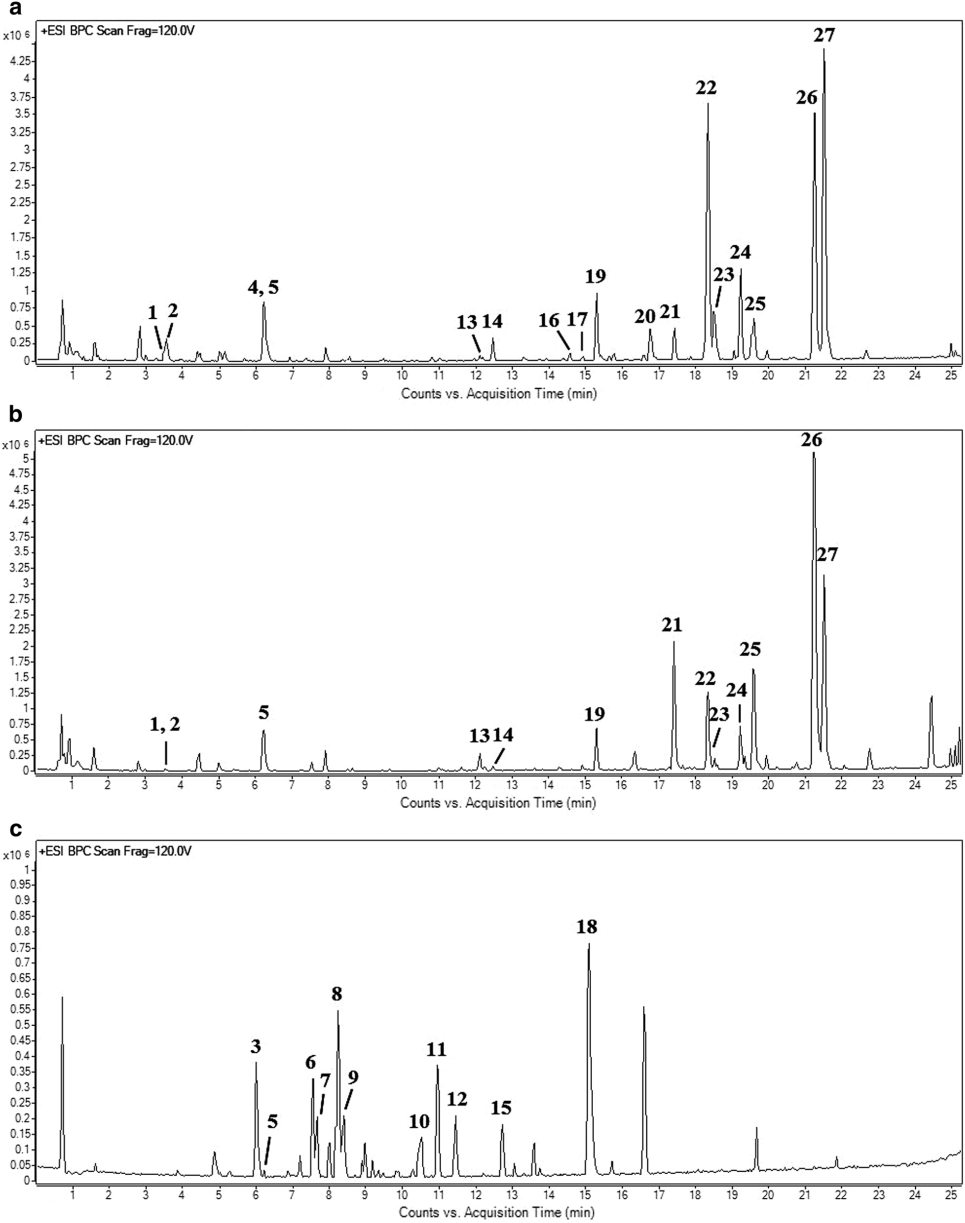
Typical BPC chromatograms of **a** *I. lophanthoides*, **b** *I. lophanthoides* var. *graciliflorus* and **c** *I. serra*

The UPLC-ESI-QTOF-MS/MS chromatograms revealed the chemical profiles of the three different *Isodon* species, and typical base peak chromatograms (BPC) of these three species are shown in Fig. 3. The chemical profiles of IL and ILG were similar, whereas the chemical profile of IS was considerably different. By comparing the samples of the different batches of IS, IL and IL, we were able to select 11, 17 and 13 common peaks for each species, respectively. One common peak (peak 5) was found in all three species. Thirteen common peaks were found in the chromatograms of IL and ILC, namely peaks 1, 2, 5, 13, 14, 19, 21, 22, 23, 24, 25, 26 and 27. The main difference between IL and ILG was that peaks 4, 16, 17 and 20 only appeared in IL. Moreover, peak 13 was absent or only detected in small quantities in ILG, but was distinct in IL; peak 14 was absent or only detected in small quantities in IL, but was distinct in ILG; peak 22 was present in much higher quantities in IL than ILG; and peak 27 was detected in higher quantities than peak 26 in IL, and vice versa in ILG. However, 10 specific peaks (peaks 3, 6, 7, 8, 9, 10, 11, 12, 15 and 18) were only detected in the chromatogram of IS.

**Fig. 4:**
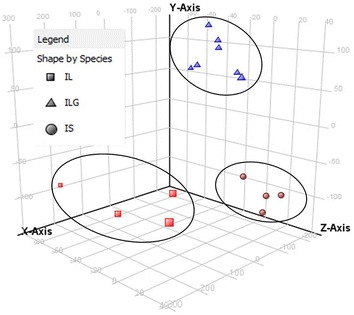
3D PCA plot of the samples

**Fig. 5:**
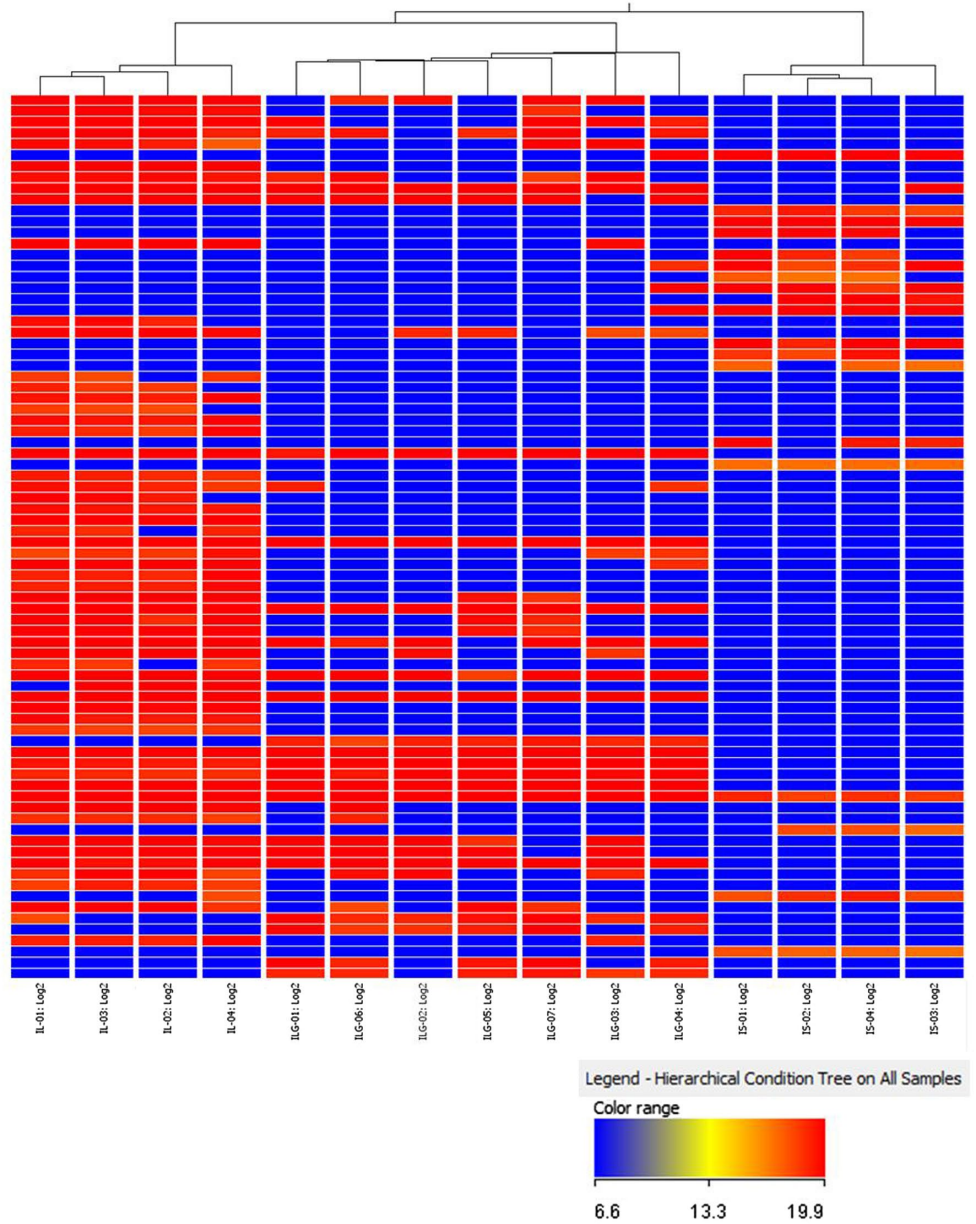
Hierarchical cluster analysis heat map for the association of compounds detected in samples. Each* line* represents an *m/z* signal detected in MS, whereas the* color* indicates the log_2_-transformed *m/z* signal intensities

All of the raw UPLC-MS data for the reference plants were examined using the Agilent MassHunter Mass Profiler Professional Software to further validate the differences between the three *Isodon* species. PCA was conducted, which exposed the variance between the samples by converting the numerous variables into principal components. The UPLC-MS data for the plants were imported for the calculation. Following the reduction of these data by statistical means, including ANOVA and the use of a frequency filter, three principal components were calculated. The PCA plot with principal components 1 (41.20%), 2 (22.26%) and 3 (7.42%) is shown in Fig. 4. The samples were distinctively clustered in three groups, which were correlated with the three species. The UPLC-MS features of the three species were different and these data could be used to distinguish between the three species. HCA was also used to interpret the relationships between the three different species. By comparing the detected *m/z* signals and signal intensities of the UPLC-MS data, samples were organized into clusters based on the similarity in their MS profiles. Figure 5 shows the dendrogram of hierarchical clustering, where the samples have been grouped into three branches correlating to the three species. These data highlighted the closer relationship between IL and ILG compared with IS. The HCA results therefore supported those of the PCA, and revealed the phylogenetic relationships between the three *Isodon* species.

**Fig. 6:**
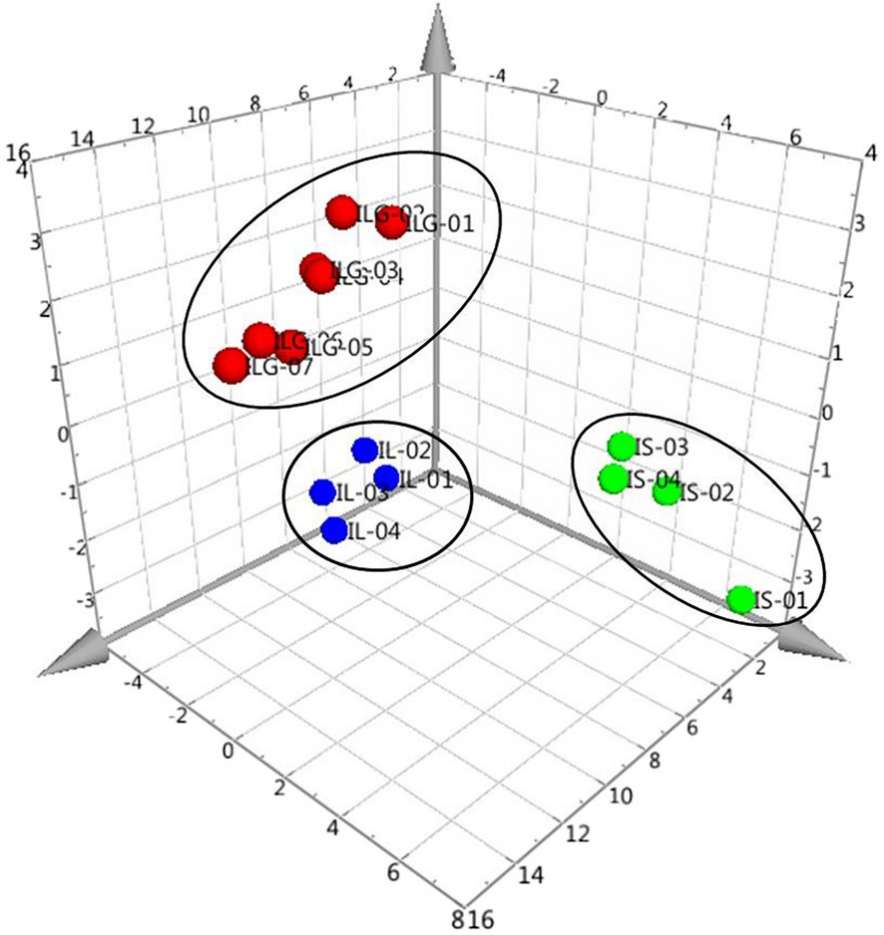
The OPLS-DA plot of samples using the selected peaks

Twenty-seven different peaks were selected from the chromatograms of the three different species to allow for their differentiation. Furthermore, the ability of these peaks to distinguish between the different species was verified by OPLS-DA. A 3D OPLS-DA is shown in Fig. 6, where the samples were separated into three groups by species. The consistency observed between these results and those obtained using the MS data highlighted the representative nature of the selected peaks.

### Peak assignments and the interpretation of MS/MS spectra

**Fig. 7:**
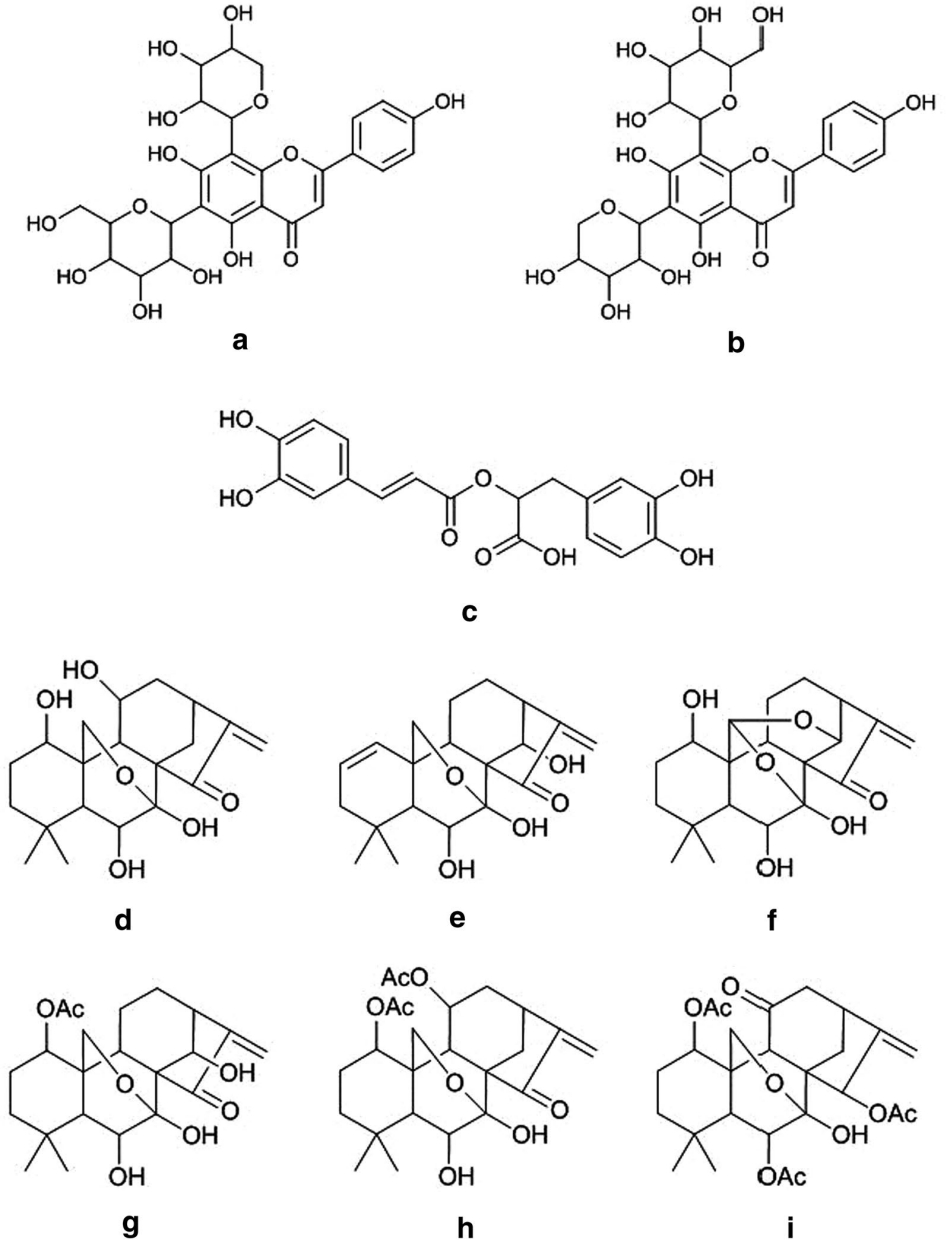
Chemical structures of the identified compounds. **a** Schaftoside; **b** Isoschaftoside; **c** Rosmarinic acid; **d** Lasiodonin; **e** Oridonin; **f** Ponicidin; **g** Lasiokaurin; **h** Shikokianin; **i** Shikokianidin

Peak assignments for the three species were conducted according to the references, standard compounds and possible fragmentation pathways. The details of the peaks identified from the three *Isodon* species are summarized in Table 3, and the structures of the identified compounds are shown in Fig. 7.

**Table 3:** Identification information of the selected peaks

Peak no.	Retention time (min)	MS^1^ m/z (relative abundance)	MS^2^ m/z of the molecular ion (relative abundance)	Identity/classification	Calculated formula	Calculated mass	Mass accuracy (ppm)
1	3.47	587.1347 [M+Na]^+^ (30), 565.1537 [M+H]^+^ (100)	565.1522 [M+H]^+^(59), 547.1419 [M+H–H_2_O]^+^(100), 529.1325 [M+H–2H_2_O]^+^(47), 511.1227 [M+H–3H_2_O]^+^(28), 499.1208 [M+H–2H_2_O–CH_2_O]^+^(42), 493.1086 [M+H–4H_2_O]^+^(13), 481.1106 [M+H–3H_2_O–CH_2_O]^+^(17), 469.1112 [M+H–2H_2_O–C_2_H_4_O_2_]^+^(31), 457.1105 [M+H–H_2_O–C_3_H_6_O_3_]^+^(33), 445.1118 [M+H–5H_2_O–CH_2_O]^+^(40)	Schaftoside	C_26_H_28_O_14_	564.1479	−2.66
2	3.54	587.1347 [M+Na]^+^ (24), 565.1538 [M+H]^+^ (100)	565.1538 [M+H]^+^(100), 547.1428 [M+H–H_2_O]^+^(71). 529.1332 [M+H–2H_2_O]^+^(64), 511.1213[M+H–3H_2_O]^+^ (56), 499.1217 [M+H–2H_2_O–CH_2_O]^+^(28), 481.1108 [M+H–3H_2_O–CH_2_O]^+^(17), 445.1111 [M+H–5H_2_O–CH_2_O]^+^(14), 427.1012 [M+H–H_2_O–C_4_H_8_O_4_]^+^(86), 409.0906 [M+H–2H_2_O–C_4_H_8_O_4_]^+^(38)	Isoschaftoside	C_26_H_28_O_14_	564.1479	−2.48
3	5.98	387.1765 [M+Na]^+^ (100), 365.1949 [M+H]^+^ (70), 347.1843 [M+H–H_2_O]^+^ (35)	347.1843 [M+H–H_2_O]^+^ (100), 329.1744 [M+H–2H_2_O]^+^(46), 311.1631 [M+H–3H_2_O]^+^ (84), 299.1637 [M+H–2H_2_O–CH_2_O]^+^(64), 283.1685 [M+H–3H_2_O–CO]^+^(37), 281.1524 [M+H–3H_2_O–CH_2_O]^+^(61), 265.1568 [M+H–4H_2_O–CO]^+^(21), 253.1568 [M+H–3H_2_O–CH_2_O–CO]^+^(14)	Lasiodonin	C_20_H_28_O_6_	364.1886	−2.75
4	6.21	629.1450 [M+Na]^+^ (2), 607.1645 [M+H]^+^ (100)	607.1634[M+H]^+^ (100), 589.1527 [M+H–H_2_O]^+^(54), 571.1417 [M+H–2H_2_O]^+^(35), 553.1315 [M+H–3H_2_O]^+^(29), 541.1323 [M+H–2H_2_O–CH_2_O]^+^(14), 523.1222[M+H–3H_2_O–CH_2_O]^+^(13), 469.1110 [M+H–H_2_O–C_4_H_8_O_4_]^+^ (12), 427.1011 [M+H–2H_2_O–C_2_H_4_O_2_]^+^(45), 409.0902 [M+H–3H_2_O–C_2_H_4_O_2_]^+^ (22)	Glycoside	C_28_H_30_O_15_	606.1584	−1.48
5	6.22	383.0724 [M+Na]^+^ (20), 343.0805 [M+H–H_2_O]^+^ (11), 163.0385 [M+H–C_9_H_10_O_5_]^+^ (100)	163.0385 [M+H–C_9_H_10_O_5_]^+^(100), 145.0287 [M+H–C_9_H_12_O_6_]^+^(6)^a^	Rosmarinic acid	C_18_H_16_O_8_	360.0845	−1.94
6	7.55	387.1765 [M+Na]^+^ (100), 365.1950 [M+H]^+^ (49), 347.1841 [M+H–H_2_O]^+^ (22)	347.1838 [M+H–H_2_O]^+^(16), 329.1739 [M+H–2H_2_O]^+^(30), 311.1631 [M+H–3H_2_O]^+^(100), 299.1637 [M+H–2H_2_O–CH_2_O]^+^(23), 283.1680 [M+H–3H_2_O–CO]^+^(23), 281.1528 [M+H–3H_2_O–CH_2_O]^+^(30)	C-20 oxygenated *ent*-kaurane diterpenoids	C_20_H_28_O_6_	364.1886	−2.47
7	7.67	387.1771 [M+Na]^+^ (100), 365.1942 [M+H]^+^ (7), 347.1847 [M+H–H_2_O]^+^ (73)	347.1834 [M+H–H_2_O]^+^(14), 329.1740 [M+H–2H_2_O]^+^(22), 311.1630 [M+H–3H_2_O]^+^ (20), 301.1789 [M+H–2H_2_O–CO]^+^(100), 299.1630 [M+H–2H_2_O–CH_2_O]^+^(23), 283.1685 [M+H–3H_2_O–CO]^+^(68), 281.1528 [M+H–3H_2_O–CH_2_O]^+^(59)^a^	Oridonin	C_20_H_28_O_6_	364.1886	−1.92
8	8.27	747.3332 [2 M+Na]^+^ (100), 385.1610 [M+Na]^+^ (85), 363.1794 [M+H]^+^ (44)	363.1788 [M+H]^+^(18), 345.1686 [M+H–H_2_O]^+^(32), 327.1577 [M+H–2H_2_O]^+^(58), 309.1490 [M+H–3H_2_O]^+^(25), 299.1627 [M+H–2H_2_O–CO]^+^(73), 281.1529 [M+H–2H_2_O–COOH]^+^(100), 253.1569 [M+H–2H_2_O–COOH–CO]^+^(20)^a^	Ponicidin	C_20_H_26_O_6_	362.1729	−1.10
9	8.39	385.1613 [M+Na]^+^ (100), 367.1510 [M–H_2_O + Na]^+^ (11), 345.1688 [M+H–H_2_O]^+^ (52)	345.1681 [M+H–H_2_O]^+^(100), 327.1576 [M+H–2H_2_O]^+^(13), 309.1474 [M+H–3H_2_O]^+^(35), 299.1620 [M+H–2H_2_O–CO]^+^(23), 281.1530 [M+H–2H_2_O–COOH]^+^(67), 271.1678 [M+H–2H_2_O–2CO]^+^(15), 253.1559 [M+H–2H_2_O–COOH–CO]^+^(12)	*ent*-kaurane diterpenoids	C_20_H_26_O_6_	362.1729	−2.21
10	10.41	429.1878 [M+Na]^+^ (100), 407.2065 [M+H]^+^ (23), 389.1957 [M+H–H_2_O]^+^ (3)	389.1956 [M+H–H_2_O]^+^(18), 329.1725 [M+H–H_2_O–AcOH]^+^(36), 311.1162 [M+H–2H_2_O–AcOH]^+^(13), 299.1629 [M+H–H_2_O–AcOH–CH_2_O]^+^ (100), 281.1529 [M+H–2H_2_O–AcOH–CH_2_O]^+^ (30),253.1572 [M+H–2H_2_O–AcOH–CH_2_O–CO]^+^(45)	Lasiokaurin	C_22_H_30_O_7_	406.1991	0.25
11	10.96	429.1875 [M+Na]^+^ (100), 407.2058 [M+H]^+^ (3), 389.1956 [M+H–H_2_O]^+^ (42)	371.8261 [M+H–2H_2_O]^+^(14), 343.1874 [M+H–2H_2_O–CO]^+^ (11), 329.1751 [M+H–H_2_O–AcOH]^+^(85), 311.1630 [M+H–2H_2_O–AcOH]^+^(64), 299.1615 M+H–H_2_O–AcOH–CH_2_O]^+^(72), 283.1686 [M+H–2H_2_O–AcOH–CO]^+^(89), 281.1526 [M+H–2H_2_O–AcOH–CH_2_O]^+^(100), 265.1580 [M+H–3H_2_O–AcOH–CO]^+^(46), 253.1576 [M+H–2H_2_O–AcOH–CH_2_O–CO]^+^ (61)	C-20 oxygenated *ent*-kaurane diterpenoids	C_22_H_30_O_7_	406.1991	−1.97
12	11.47	471.1983 [M+Na]^+^ (100), 449.2159 [M+H]^+^ (47)	389.1948 [M+H–AcOH]^+^(12), 371.1841 [M+H–H_2_O–AcOH]^+^(21), 329.1743 [M+H–2AcOH]^+^(22), 311.1633 [M+H–H_2_O–2AcOH]^+^(100), 293.1531 [M+H–2H_2_O–2AcOH]^+^(15), 283.1688 [M+H–H_2_O–2AcOH–CO]^+^(24), 281.1527 [M+H–H_2_O–2AcOH–CH_2_O]^+^(66)	Shikokianin	C_24_H_32_O_8_	448.2097	−2.45
13	12.07	353.1714 [M+Na]^+^ (100), 313.1794 [M+H–H_2_O]^+^ (14)	295.1669 [M+H–2H_2_O]^+^ (4), 277.1590 [M+H–3H_2_O]^+^(23), 237.1273 [M+H–3H_2_O–C_3_H_4_]^+^(100), 209.0948 [M+H–3H_2_O–C_5_H_8_] (17)	Abietane diterpenoids	C_20_H_26_O_4_	330.1831	−2.73
14	12.45	353.1714 [M+Na]^+^ (38), 331.1889 [M+H]^+^ (4), 313.1798 [M+H–H_2_O]^+^ (100)	253.1219 [M+H–2H_2_O–C_3_H_6_]^+^(9), 227.1056 [M+H–2H_2_O–C_5_H_8_]^+^(100), 212.0822[M+H–2H_2_O–C_5_H_8_–CH_3_]^+^(14), 209.0955 [M+H–3H_2_O–C_5_H_8_]^+^(16), 199.1114 M+H–2H_2_O–C_5_H_8_–CO]^+^(21)	Abietane diterpenoids	C_20_H_26_O_4_	330.1831	−2.73
15	12.73	413.1925 [M+Na]^+^ (100), 391.2103 [M+H]^+^ (16), 373.1998 [M+H–H_2_O]^+^ (10)	391.2000 [M+H]^+^(6), 373.2000 [M+H–H_2_O]^+^(50), 313.1781 [M+H–H_2_O–AcOH]^+^(66), 295.1673 [M+H–2H_2_O–AcOH]^+^(50), 283.1678 [M+H–H_2_O–AcOH–CH_2_O]^+^(100), 267.1730 [M+H–2H_2_O–AcOH–CO]^+^(57), 265.1576 [M+H–2H_2_O–AcOH–CH_2_O]^+^(22)	C-20 oxygenated *ent*-kaurane diterpenoids	C_22_H_30_O_6_	390.2042	-2.31
16	14.53	417.2266 [M+H]^+^ (100), 357.2056 [M+H–AcOH]^+^ (91)	315.1940 [M+H–AcOH–CH_2_=CO]^+^(100), 297.1841 [M+H–2AcOH]^+^(75), 279.1726 [M+H–H_2_O–2AcOH]^+^(26), 255.1369 [M+H–2AcOH–C_3_H_6_]^+^(48), 229.1214 [M+H–2AcOH–C_5_H_8_]^+^(26)	Abietane diterpenoids	C_24_H_32_O_6_	416.2199	−1.44
17	14.89	375.2161 [M+H]^+^ (100), 315.1947 [M+H–AcOH]^+^ (27)	315.1946 [M+H–AcOH]^+^(87), 297.1846 [M+H–H_2_O–AcOH]^+^(68), 287.1998 [M+H–AcOH–CO]^+^(22), 279.1723 [M+H–2H_2_O–AcOH]^+^(13), 273.1468 [M+H–AcOH–C_3_H_6_](16), 255.1369 [M+H–3H_2_O–AcOH]^+^(19), 229.1220 [M+H–H_2_O–AcOH–C_5_H_8_]^+^(100), 205.1215 [M+H–AcOH–C_7_H_10_O]^+^(48)	Abietane diterpenoids	C_22_H_30_O_5_	374.2093	−1.34
18	15.07	513.2076 [M+Na]^+^ (100), 508.2521 [M+NH4]^+^ (4), 491.2253 [M+H]^+^ (3)	431.2025 [M+H–AcOH]^+^(8), 389.1976 [M+H–AcOH–CH_2_=CO]^+^(22), 371.1830 [M+H–2AcOH]^+^(49), 329.1740 [M+H–2AcOH–CH_2_=CO]^+^(100), 311.1634 [M+H–H_2_O–2AcOH–CH_2_=CO]^+^(38), 283.1708 [M+H–H_2_O–2AcOH–CH_2_=CO–CO]^+^(29)^b^	Shikokianidin	C_26_H_34_O_9_	490.2203	−3.88
19	15.28	411.1765 [M+Na]^+^ (17), 389.1946 [M+H]^+^ (100)	371.1847 [M+H–H_2_O]^+^(15), 311.1633[M+H–H_2_O–AcOH]^+^ (38), 269.1164 [M+H–H_2_O–AcOH–C_3_H_6_]^+^ (34), 243.1015 [M+H–H_2_O–AcOH–C_5_H_8_]^+^(100), 225.0903[M+H–2H_2_O–AcOH–C_5_H_8_]^+^ (11)	Abietane diterpenoids	C_22_H_28_O_6_	388.1886	−3.35
20	16.78	455.2022 [M+Na]^+^ (13), 433.2203 [M+H]^+^ (100)	331.1889 [M+H–AcOH–CH_2_=CO]^+^(19), 313.1785 [M+H–2AcOH]^+^(100), 271.1332 [M+H–2AcOH–C_3_H_6_]^+^(13), 245.1164 [M+H–2AcOH–C_5_H_8_]^+^(19), 219.1012[M+H–2AcOH–C_7_H_10_]^+^(37), 205.0855[M+H–2AcOH–C_8_H_12_]^+^ (37)	Abietane diterpenoids	C_24_H_32_O_7_	432.2148	−4.16
21	17.42	353.1716 [M+Na]^+^ (22), 331.1894 [M+H]^+^ (100)	313.1793 [M+H–H_2_O]^+^(100), 257.1168 [M+H–H_2_O–C_4_H_8_]^+^(11), 243.1014 [M+H–H_2_O–C_5_H_10_]^+^(74)	Abietane diterpenoids	C_20_H_26_O_4_	330.1831	−2.12
22	18.32	395.1816 [M+Na]^+^ (26), 373.2015 [M+H]^+^ (100), 355.1896 [M+H–H_2_O]^+^ (10)	355.1892 [M+H–H_2_O]^+^ (9), 337.1791 [M+H–2H_2_O]^+^(14), 295.1684 [M+H–2H_2_O–CH_2_=CO]^+^(42), 277.1578 [M+H–2H_2_O–AcOH]^+^(26), 253.1219 [M+H–2H_2_O–CH_2_=CO–C_3_H_6_]^+^(87), 239.1059 [M+H–2H_2_O–CH_2_=CO–C_4_H_8_]^+^(34), 227.1066 [M+H–2H_2_O–CH_2_=CO–C_5_H_8_]^+^(100), 211.1112 [M+H–2H_2_O–CH_2_=CO–C_4_H_8_–CO]^+^(24), 209.0956 [M+H–2H_2_O–AcOH–C_5_H_8_]^+^(50)	Abietane diterpenoids	C_22_H_28_O_5_	372.1937	1.34
23	18.52	381.2025 [M+Na]^+^ (100), 359.2210 [M+H]^+^ (20)	299.1999 [M+H–AcOH]^+^(100), 271.1680 [M+H–AcOH–C_2_H_4_]^+^(25)	Diterpenoids	C_22_H_30_O_4_	358.2144	−1.95
24	19.23	435.1745 [M + 2Na]^+^ (7), 413.1926 [M+Na]^+^ (100), 373.2005 [M+H–H_2_O]^+^ (8), 313.1793 [M+H–H_2_O–AcOH]^+^ (10)	355.1857 [M+H–2H_2_O]^+^(7), 313.1798 [M+H–H_2_O–AcOH]^+^(93), 295.1678 [M+H–2H_2_O–AcOH]^+^(25), 285.1827 [M+H–H_2_O–CO]^+^(9), 280.1427 [M+H–2H_2_O–AcOH–CH_3_]^+^(13), 271.1673 [M+H–H_2_O–AcOH–CH_2_=CO]^+^(61), 253.1232 [M+H–2H_2_O–AcOH–C_3_H_6_]^+^(44), 243.1749 (13), 227.1038 [M+H–2H_2_O–AcOH–C_5_H_8_]^+^(17)^a^	Abietane diterpenoids	C_22_H_30_O_6_	390.2042	−2.05
25	19.61	647.3333 [2 M+Na]^+^ (100), 335.1610 [M+Na]^+^ (94), 313.1794 [M+H]^+^ (17)	295.1685 [M+H–H_2_O]^+^(13), 277.1583 [M+H–2H_2_O]^+^(43), 237.1271 [M+H–2H_2_O–C_3_H_4_]^+^(100), 227.1062[M+H–H_2_O–C_5_H_8_]^+^ (25), 209.0953 [M+H–2H_2_O–C_5_H_8_]^+^(15)	Abietane diterpenoids	C_20_H_24_O_3_	312.1725	−1.28
26	21.22	337.1765 [M+Na]^+^ (40), 315.1950 [M+H]^+^ (2), 297.1856 [M+H–H_2_O]^+^ (100)	297.1843 [M+H–H_2_O]^+^(17), 279.1736 [M+H–2H_2_O]^+^ (10), 227.1061 [M+H–H_2_O–C_5_H_10_]^+^(100), 212.0830 [M+H–H_2_O–C_5_H_10_–CH_3_]^+^(18), 199.1117 [M+H–H_2_O–C_5_H_10_–CO]^+^(19)	Diterpenoids	C_20_H_26_O_3_	314.1882	−1.59
27	21.5	455.2033 [M+Na]^+^ (100), 450.2478 [M+NH4]^+^ (8)	373.1997 [M+H–AcOH]^+^ (8), 313.1788 [M+H–2AcOH]^+^(100), 295.1682 [M+H–H_2_O–2AcOH]^+^(31), 253.1201 [M+H–H_2_O–2AcOH–C_3_H_6_]^+^ (4)^b^	Abietane diterpenoids	C_24_H_32_O_7_	432.2148	−1.62

^a^Tandem MS/MS spectra of the [M+H–H_2_O]^+^ was listed as the amount of [M+H]^+^ ion is too low

^b^Tandem MS/MS spectra of the [M+NH4]^+^ was listed as the amount of [M+H]^+^ ion is too low

Peak 5 was common to all three species. The dominant mass ion for peak 5 had an *m/z* value of 163.0392, which was consistent with the MS data of rosmarinic acid resulted from the cleavage of its ester bond, weak *m/z* 383.0724 [M+Na]^+^ and *m/z* 343.0814 [M+H–H_2_O]^+^ ions also found, which provided evidence that this peak could be attributed to rosmarinic acid [12, 14].

Diterpenoids are the major active compounds in *Isodon* species [15]. More than 500 diterpenoids have been isolated from plants belonging to this genus and a number of them have been reported to exhibit strong antibacterial, anti-inflammatory and anticancer activities [15]. Six *ent*-kaurane type diterpenoids were identified from IS in this study, including lasiodonin (peak 3), oridonin (peak 7), ponicidin (peak 8), lasiokaurin (peak 10), shikokianin (peak 13) and shikokianidin (peak 18) [11, 16]. Their chemical structures are shown in Fig. 7. The loss of H_2_O (−18 Da) from a hydroxyl group, AcOH (−60 Da) or CH_2_=CO (−42 Da) from an acetate group, CO (−28 Da) from the D ring and CH_2_O (−30 Da) from the C-20 oxygenated B ring were identified as the dominant fragmentation processes in the tandem MS/MS spectra of these compounds.

Abietane and tricyclic diterpenoids have been reported to be the major diterpenoids found in IL and ILG. Diterpenoids of this type have only ever been found in a few *Isodon* species, including *I. grandifolia* and *I. forrestii* [17]. Information pertaining to diterpenoids of this type is therefore limited. Based on the fragmentation pattern of salvialeriafone, which is a diterpenoid found in the genus *Salvia* with a similar skeletal structure to those found in IL and ILG, the proposed fragmentation of the diterpenoids was the loss of CO (−28 Da) and H_2_O (−18 Da) from their C ring after the removal of their side chains or other functional groups [18]. Although more than thirty diterpenoids were isolated from IL, ILG, *I. lophanthoides* var. *gerardianus* and *I. lophanthoides* var. *micranthus* [6, 8–10, 19], none of them showed identical molecular weight and possible fragmentation pathways to the peaks in this study. However, the calculated formulae and MS/MS fragments –C_3_H_4_ (−40 Da), –C_3_H_6_ (−42 Da) and –C_5_H_8_ (−68 Da) (removal of side chain from C ring) indicated that most of the peaks could be attributed to abietane-type diterpenoids. Furthermore, two of the peaks in the chromatograms for IL and ILG were identified as glycosides schaftoside (peak 1) and isoschaftoside (peak 2) based on comparisons with standards and references [13, 20].

### Identification of commercial samples

Based on the UPLC-MS data generated using the reference plants, we proceeded to investigate the identification of the 27 batches of commercial *Xihuangcao*. Fundamental morphologic and microscopic identification of *Xihuangcao* and several related species were done in our previous study. In terms of the morphological features of the 27 samples evaluated in this study, eight of the samples (samples 03, 04, 06, 09, 11, 14, 15, and 20) obviously did not contain plants belonging to the genus *Isodon*, which have white powders on their surface and leaves 1- or 2-pinnate (Fig. 2d). The microscopic features of these samples were also different from those of the *Isodon* species according to our preliminary studies. These samples were therefore identified as *Ampelopsis grossedentata* (Hand.-Mazz.), which is a common adulterant of *Xihuangcao* [21]. Preliminary analysis of *A. grossedentata* by UPLC-MS showed its chemical composition differed considerably from those of the *Isodon* species (Additional file 1: Figure S1), hence the UPLC-MS data for these eight batches were excluded from the automated sample class prediction.

**Fig. 8:**
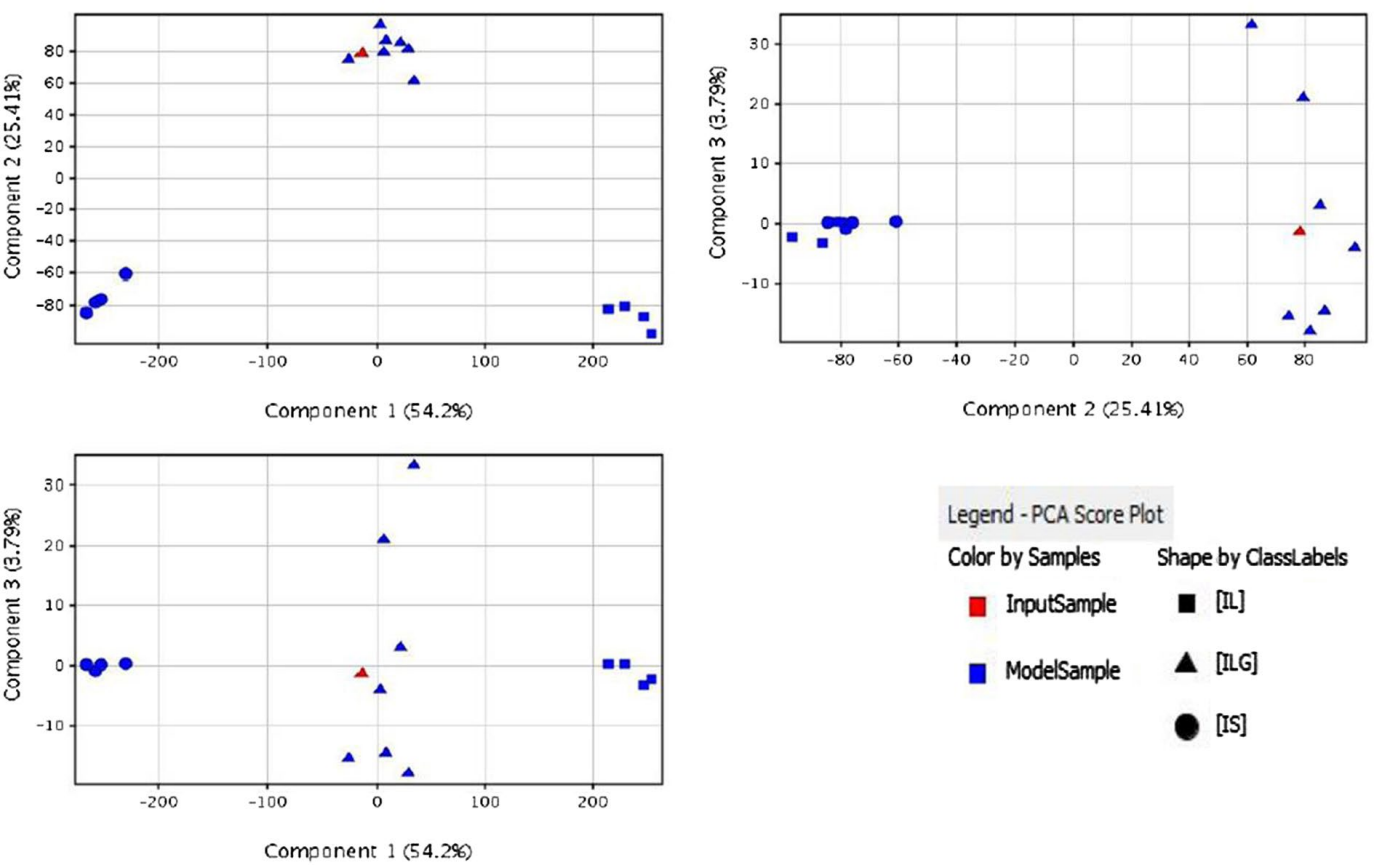
Prediction report of sample 02

A prediction model was initially built using the UPLC-MS data generated form the 15 reference plants. Significant signals were identified via a series of statistical calculations and filtration processes, followed by a statistical validation step, which showed the accuracy of the prediction for each class. The model was finally visualized by PCA. In this model, naive Bayesian classifier, which is a multi-class parameter-based statistical classifier, was used. The UPLC-MS data of the remaining 19 commercial samples were imported and classified using the prediction model. Twelve samples were predicted as ILG (samples 01, 02, 08, 13, 16, 19, 21, 22, 23, 24, 25 and 27), whereas six samples were predicted as IS (samples 05, 07, 10, 12, 18 and 26) with a high confidence measure of 1.000 in both cases. The only major exception was sample 17, which was predicted to be IS with a low confidence measure of 0.692. A representative prediction report is shown in Fig. 8. The UPLC-MS data for this sample was converted into three principal components and compared with those of the model. Manual identification of the samples using the 27 characteristic peaks was also conducted to further confirm the result. All of these results were consistent with those of the automated prediction, except for sample 17, which showed a low confidence measure. This sample could not be identified as one of the three *Isodon* species.

According to the species identification results, ILG was the major source of *Xihuangcao*, whereas IS was found in approximately one-quarter of the samples (22%) and IL was not used at all. *Xihuangcao* is mainly sold as a special local product in the markets for the preparation of soup or herbal tea. IS has a very bitter taste, whereas IL and ILG are only slightly bitter, which could explain why IS was found in small quantities in the *Xihuangcao* samples in this market investigation. IL is an annual or short-lived perennial herb, whereas its variety ILG is an undershrub capable of reaching more than 1 m in height [3]. ILG is therefore more desirable for cultivation in terms of its production yield. The large proportion of adulterant (44%) found in this study also raises concerns about the quality of the *Xihuangcao* being sold in retail markets.

## Conclusion

UPLC-ESI-QTOF-MS and subsequent chemometrics analysis were demonstrated effective to differentiate among the three different species of plants used as *Xihuangcao*.

## Additional file

**Additional file 1: Figure S1.** The BPC chromatograms of *Ampelopsis grossedentata.*

## Abbreviations

BPC: base peak chromatogram
HCA: hierarchical cluster analysis
IL: *Isodon lophanthoides*
ILG: *Isodon lophanthoides* var. *graciliflorus*
IS: *Isodon serra*
MS: mass spectrometry
ANOVA: one-way analysis of variance
OPLS-DA: orthogonal partial least squared discriminant analysis
PCA: principal component analysis
UPLC: ultra-performance liquid chromatography
UPLC-ESI-QTOF-MS: ultra-performance liquid chromatography coupled with electrospray ionization quadrupole time-of-flight mass spectrometry
