# Supplementary figures and images for: Rapid differentiation of Xihuangcao from the three Isodon species by UPLC-ESI-QTOF-MS/MS and chemometrics analysis
Source: Chin Med. 2016 Dec 15;11:48. doi: 10.1186/s13020-016-0120-y (PMC5160003; doi:10.1186/s13020-016-0120-y)

## Additional file 1

**The BPC chromatograms of *Ampelopsis grossedentata*.**

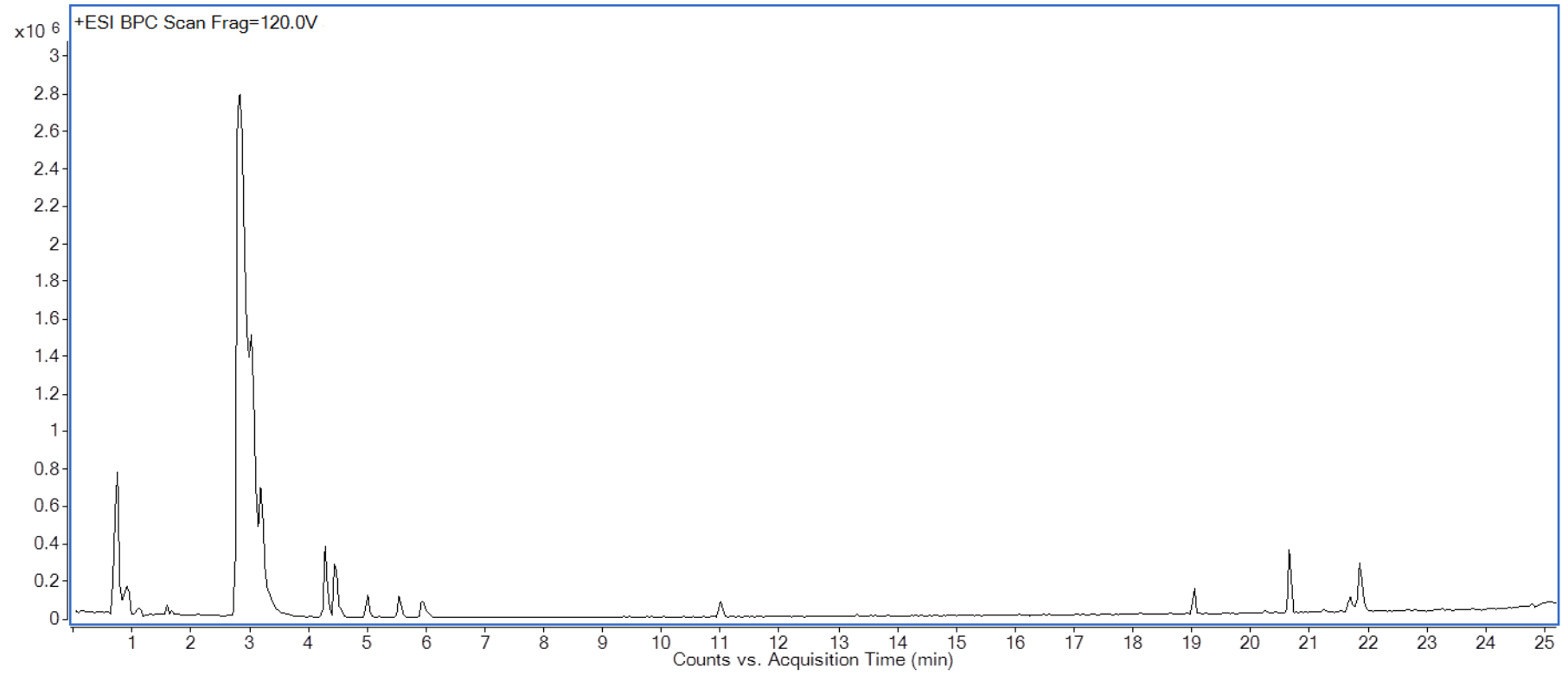

Supplement: Supplementary file 1 — Additional file 1: Figure S1. The BPC chromatograms of Ampelopsis grossedentata. [file 13020_2016_120_MOESM1_ESM.pdf]
